# The Effect of Ambient Temperature on Migraine Disease: A Scoping Review

**DOI:** 10.1002/brb3.70708

**Published:** 2025-08-21

**Authors:** James Kelbert, Joshua A. Tobin

**Affiliations:** ^1^ University of Arizona College of Medicine–Phoenix Phoenix Arizona USA; ^2^ Department of Neurology Banner University Medical Center Phoenix Arizona USA

**Keywords:** ambient temperature, migraine disease, migraine, people with migraine

## Abstract

**Introduction:**

Ambient temperature changes are hypothesized to affect migraine attacks, but few published primary articles assess such hypotheses. The authors examine the current germane literature and suggest further research.

**Methods:**

A literature search was conducted on September 23, 2023, in PubMed, Embase, and Scopus using the search terms temperature [ti] AND (headach*[ti] OR migrain*[ti]). Rayyan was utilized for duplicate detection and removal and for abstract screening. Conflicting assessments of records were resolved by consensus, and full text analysis was performed. Data was extracted by hand and tabulated.

**Results:**

Four studies met the inclusion criteria, with an additional eight found through citation analysis that analyzed tens of thousands of patients with migraine disease overall. Six studies demonstrated an association between temperature or temperature changes and migraine disease. One study identified individuals whose migraine attacks were temperature sensitive, but the association was lost when examining the whole population. The remaining five did not find any significant relationship. Every study examining patients on an individual level found a relationship between temperature and migraine disease. Studies in colder geographic areas had a greater propensity to identify cold as a trigger.

**Conclusion:**

Current data are conflicting. Temperature may be a migraine disease trigger in a subgroup of people with migraine. Lower temperatures may trigger more migraine attacks in colder climates within a subset of people with migraine. Uncontrollable factors such as air pollution, barometric pressure, and humidity are confounding variables that impede such research. Additional studies could include indoor temperature or thermostat settings during the day and night to further stratify the effects of temperature.

## Graphical abstract

This scoping review demonstrates that there may be individual‐level associations between migraine and ambient temperature in those who are temperature sensitive. This abstract was made using BioRender.

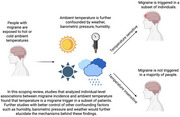

**Abbreviations**
PRISMA: preferred reporting items for systematic reviews and meta‐analyses.

## 1 Introduction

Migraine disease is a common disorder with high associated morbidity (Szok et al. 2023). It is the second leading cause of disability worldwide and affects 12% of the total population (Aguilar‐Shea et al. 2022). Its more severe version, chronic migraine disease, affects 1% to 2% of the population (Martinelli et al. 2022).

Numerous purported environmental migraine attack triggers exist, including weather change (Kelman 2007; Martinelli et al. 2022), but the evidence is inconsistent. Using a semi‐structured interview, Wöber et al. (2006) reported that 83% of patients reported weather as a migraine attack trigger. A meta‐analysis of migraine disease triggers noted five studies in which hot weather was reported as a trigger, seven studies in which cold weather was a reported trigger, and nine studies in which heat was a reported trigger (Pellegrino et al. 2018). However, a key inclusion criterion was that data was retrospectively self‐reported, which could lead to confounding memory bias.

Pelzer et al. (2023) hypothesized that disrupted brain internal thermoregulation triggers migraine attacks but noted that there is no way to systematically measure brain temperature in people with migraine. Nonetheless, several potential mechanisms of action exist whereby ambient temperature changes could trigger migraine disease.

There has not been an aggregate appraisal of evidence to date to determine the overall accuracy of patient‐reported events throughout the literature. In other words, the relationship between ambient temperature and migraine disease has thus far been investigated primarily in the context of weather, and there is no systematic assessment of the relationship between temperature and migraine attacks (Becker 2011). This scoping review aims to address that discrepancy.

## 2 Methodology

### 2.1 Literature Search and Paper Selection

This scoping review was conducted following Preferred Reporting Items for Systematic Reviews and Meta‐analyses (PRISMA) guidelines (Page et al. 2021). Institutional Review Board approval was waived, given that this is a review article and does not directly involve any personal health information or patient data. A literature search was conducted on September 23, 2023, in PubMed, Embase, and Scopus. Search terms were as follows: temperature [ti] AND (headach*[ti] OR migrain*[ti]) for PubMed, temperature:ti AND (headach*:ti OR migrain*:ti) for Embase, and TITLE(migraine OR headache) AND TITLE(temperature) for Scopus.

No additional search parameters or filters were applied. Article abstracts were uploaded to the systematic review software Rayyan (Ouzzani et al. 2016). Duplicates of records were removed prior to the screening process. Titles and abstracts of the remaining records were screened by the other reviewer. Conflicting assessments of records were resolved by consensus.

Inclusion criteria were studies involving patients diagnosed with episodic migraine disease, studies reporting any relationship of ambient temperature, studies in the English language, and studies reporting adult patients at least 18 years of age. In the case where a study involved patient populations with migraine disease and another headache disorder, the paper was included. This decision was ultimately made because there is a scarcity of literature investigating this relationship, and the inclusion of primary material that limits specificity was determined to be preferable to omitting evidence. When possible, a focused analysis of these papers on people with migraine was conducted. The exclusion criteria were animal studies, pediatric populations, studies not reported in English, and abstracts, case reports, case series, letters to the editor, editorials, meta‐analyses, and review studies. To maximize the chances of identifying all relevant studies, the reference lists of studies were manually checked to perform forward and backward citation analyses.

### 2.2 Data Extraction

Articles included in the abstract screening stage were exported into Excel. From each paper, the following information was extracted: year(s) studies were conducted, study type, level of evidence, sample size, age range of participants, patient demographics, temperature data investigated, migraine disease incidence, and whether individual or group temperature data was assessed. Given the paucity of comparable information, statistical analysis was not performed.

## 3 Results

**FIGURE 1:**
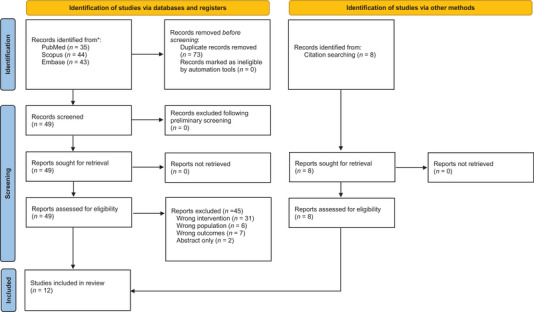
Preferred Reporting in Systematic Reviews and Meta Analyses (PRISMA) flowchart for all included studies.

Of the 122 total results from the search, 73 were duplicates. Of the 49 remaining, 6 studied the wrong population, 31 used the wrong intervention, 7 assessed the wrong outcomes, and 2 were abstracts, leaving 4 that met all inclusion criteria (Figure 1). From those 4, 8 additional studies were found via backward and forward citation analyses, leaving a total of 12 included articles (Table 1).

**TABLE 1:** Description of demographics and findings of all included articles.

Article	Population	Sample size (% female)	Study design	Migraine data available	Temperature data available	Key finding	Individual temperature assessment?
Gomersall and Stuart (1973)	People with migraine living within 10 miles of Aberdeen, Scotland, recruited via the British Migraine Association and the local press	56	Prospective case series	Documentation of each headache with time of onset, duration, severity, and associated features. November 1969–May 1970	Local meteorology station	There was no relationship between migraine attacks and temperature.	No
Wilkinson and Woodrow (1979)	Patients attending the Princess Margaret Migraine Clinic in London	310	Retrospective case series	time of onset of migraine	Temperature change from 3 h prior to the onset of the headache attack to the temperature at the time of the onset of the headache attack.	There was no relationship between migraine attacks and temperature.	No
De Matteis et al. (1994)	Patients in a “small geographic area,” presumably near the University of L'Aquila, Italy, attending a headache clinic.	40 (62.5%)	Prospective cross‐sectional	Headache diary from March to June 1988	mean daily temperature value	No significant correlation between maximum daily temperature and migraine frequency with no given temperature range	No
Villeneuve et al. (2006)	Civic Hospital, Ottawa, serving a population generally residing within 40 km	4051 (74%)	Prospective case‐crossover	presentation to ER for migraine from 1993 to 2000	Hourly, recorded at an airport 15 km south of the ER	There was no statistically significant association with temperature or temperature changes in the number of ER visits for migraines	No
Mukamal et al. (2009)	patients living within 40 km of and seen at Beth Israel Deaconess Medical Center ED, Boston, for migraine	7054 (75%)	Prospective case‐crossover	Time of onset of migraine from 2000 to 2007	Hourly surface observations of the National Weather Service First Order Station at Logan Airport (East Boston).	Higher mean ambient temperature in the 24 h preceding hospital presentation increased the acute risk of headache	No
Zebenholzer et al. (2011)	patients living within 25 km of the Vienna meteorological station	238 (87.8%)	Prospective case series	Headache dairy completed between October 1, 2002 and March 31, 2003.	Daily average, maximum, and minimum temperatures from a recording station in the same city	There was no relationship between migraine attacks and temperature.	No
Hoffmann et al. (2011)	people with episodic migraine attacks attending a headache clinic in Berlin	20 (75%)	Retrospective case series	headache diary data over 12 consecutive months between January 2006 and December 2007 with data in 4‐hour intervals	Temperature provided by the German Meteorological Service and analyzed in 4‐hour intervals.	In a subset of patients, lower temperature and higher relative humidity correlate with the onset of a migraine period independently of the time of the day	Yes
Scheidt et al. (2013)	People with migraine living in Germany	Not stated; total 4720 migraine messages (68.4%)	Prospective cross‐sectional	Documentation of migraine onset in an electronic headache diary or web form from June 2011 to February 2012	German Meteorological Service mean daily temperature at the station closest to each patient	Increase in temperature associated with increase in migraine messages sent	No
Hoffmann et al. (2015)	People with migraine living within 50 km of Berlin and attending a headache clinic on a stable preventive regimen	100	Retrospective case series	Same as Hoffmann et al. (2011)	Same as Hoffmann et al. (2011)	Absolute temperature and temperature increase were migraine triggers for select people with migraine	Yes
Yilmaz et al. (2015)	Admitted to a regional hospital ED in Turkey with a diagnosis of migraine between January 1 and December 31, 2013	3491 (72%)	Retrospective case series	Time of onset of migraine	Daily average, maximum, and minimum temperatures from a recording station in the same city	Increased number of patients with migraine on days with higher daily maximum temperature	No
Yang et al. (2015)	City of Taipei	66 (75.8%)	Prospective cohort	7‐month headache diary from January to Decemner 2007	Daily temperature data for Taipei provided by the Central Weather Bureau, Taiwan	Temperature fluctuations accounted for a proportion of migraine incidence in winter and summer	No
Lee et al. (2018)	City of Seoul	18921 (72%)	Retrospective Database Inquiry	Patients who presented to emergency departments for migraine	Hourly, from one weather monitoring station “slightly north of the center of the city” operated by the Korea Meteorological Administration	Temperatures above the 75th percentile increased the effect of air pollution as a migraine trigger by 18% in migraine without aura.	No

### 3.1 Studies With an Association Between Temperature and Migraine Disease

In Scheidt et al., people with migraine disease sent a message to the research team whenever they experienced a migraine attack. They found that a 5°C decrease 1–3 days prior was associated with a 24 ± 8% increase in migraine disease messages, whereas a 5°C increase was associated with a 19 ± 7% increase in migraine disease messages. Both changes were statistically significant. There was no significant difference in message frequency 1–3 days after said mean temperature change (Scheidt et al. 2013).

Hoffmann et al. (2011) noted lower temperatures 18 and 24 h prior to the onset of a new migraine attack period, defined as one or more migraine attacks separated by less than 24 h. While the group's subsequent 2015 study did not identify an association for the population as a whole (Hoffmann et al. 2015), both studies identified individuals for whom temperature was a trigger. For the combined population of both studies, high or increasing temperature predicted migraine attacks at 5 time points (all in the 2011 article) and 12 patient time points prior to attack onset, whereas low or decreasing temperature predicted migraine attacks in 12 patients (10 in the 2011 article and 2 in the 2015 article) and 32 patient time points.

Yang et al. found that temperature fluctuations explained 29.2% of the variance in headache attack incidence during winter and 6.0% during spring for people with migraine who perceived themselves as temperature sensitive. In contrast, temperature accounted for 14.8% of the variance in headache incidence exclusively during summer for people with migraine who perceived themselves as temperature nonsensitive (Yang et al. 2015).

Mukamal et al. (2009) determined that a 5°C increase in mean ambient temperature in the 24 h prior increased the risk of ED presentation for migraine attacks by 1.111 (1.013–1.218), and a one interquartile range increase by 1.389 (1.042–1.852).

Yilmaz et al. (2015) found significant Pearson's correlations between the daily number of patients admitted to an ED in Turkey and the daily maximum temperature (*p* = 0.005), mean temperature (*p* = 0.013), minimum temperature (*p* = 0.041), and daily temperature change (*p* = 0.003).

Lee et al. found that temperatures above the 75th percentile increased the effect of air pollution as a migraine disease trigger by 18% in migraine attacks without aura. Interactions between air pollution and temperature were shown among women (Pinteract for PM2.5 = 0.07; PM10 = 0.05; O3 = 0.09) and patients aged under 40 years (Pinteract for PM2.5 = 0.04; PM10 = 0.03; CO = 0.04). The air pollution effect in migraine attacks with or without aura was greater on high‐temperature days with statistical evidence of interaction for particles < 2.5 µm (OR 1.068 [95% CI: 1.029–1.108]) and particles < 10 µm (high: OR 1.066 [95% CI: 1.025–1.109]) (Lee et al. 2018).

### 3.2 Studies Without an Association Between Temperature and Migraine Disease

Conversely, other studies demonstrated no significant association between temperature changes and migraine. Gomersall and Stuart (1973) and Wilkinson and Woodrow (1979) found no association between migraine and weather, temperature, or humidity changes (Gomersall and Stuart 1973; Wilkinson and Woodrow 1979). De Matteis et al. (1994) found no significant correlation between maximum daily temperature and migraine frequency but did not state the temperature range they analyzed. Villeneuve et al. (2006) did not identify any relationship between absolute or changes in temperature and migraine attack incidence or severity, with odds ratios ranging from 0.99 to 1.04, ranging from < 1 to 24 h.

More recent studies with more advanced study designs and more patients also found a negligible effect of temperature. Zebenholzer et al. found a negligible effect of weather on migraine disease with an odds ratio of 1.02 (*p* = 0.023, not significant after correction for multiple testing) for migraine given day‐to‐day change of minimum air temperature. A big data analysis from Hoffmann et al. (2015) underscored that only a subgroup of people with migraine (7% of the total population), but not the population as a whole, was sensitive to absolute changes in temperature.

## 4 Discussion

Temperature change may trigger migraine through several molecular mechanisms. For example, the short‐chain fatty acids butanoate and propionate are produced in the colon via bacterial fermentation of dietary fibers (Zhang et al. 2023). Administration of either butanoate or propionate attenuated hyperalgesia and pain in a mouse migraine model (Lanza et al. 2021, 2022). Yi et al. (2021) found that low temperature increased propionate levels, whereas high temperature decreased butyrate levels, suggesting two mechanisms whereby heat could trigger migraine. Sugar metabolism may also play a role. Fasting and reactive hypoglycemia following large carbohydrate intakes are known migraine triggers, and ambient temperatures less than 10°C and greater than 20°C were associated with an increased frequency of hypoglycemia compared to 10°C–20°C (Hensel et al. 2017; Hufnagl and Peroutka 2002).

Given the high prevalence and associated disability of migraine disease, identifying environmental triggers alongside pharmaceutical agents is crucial. This review surveys the existing literature exploring the effect of temperature or temperature changes on migraine attack incidence and severity.

Studies conducted in cooler geographic areas tended to identify more associations with cold than with heat as a trigger, though such differences either were not statistically significant or reliably quantified (Gomersall and Stuart 1973; Villeneuve et al. 2006; Zebenholzer et al. 2011). It would also be interesting to know whether temperature increases are more of a migraine attack trigger during hot weather and whether temperature decreases are more of a migraine attack trigger during cold weather, as only one study found a weak univariate correlation between maximum and minimum temperature and migraine attack incidence (Yilmaz et al. 2015).

The 12 studies included in this review suggest that a change in mean temperature may be a migraine attack trigger. This is not entirely clear, given some studies that do not report their temperature range or have small sample sizes (De Matteis et al. 1994). Yang et al. (2015) attempted to determine if temperature fluctuations were attributable to people who experience temperature‐sensitive migraines and to those who self‐classified as not. The database study by Lee et al. (2018) found that temperature fluctuations above the 75th percentile significantly increased the effect of air pollution as a migraine trigger. Scheidt et al. (2013) also demonstrated an association between temperature changes and migraine incidence.

Yang's finding that temperature changes are more of a trigger in self‐reported temperature‐sensitive patients (29.2% in one season and 6.0% in another season) than in self‐reported temperature‐insensitive patients (14.8% in only one season) is a reminder that patients tend to know themselves. However, temperature changes may be a trigger even in those who do not consider themselves temperature sensitive (Yang et al. 2015), but further investigation is needed. Synergism between air pollution and heat is consistent with previously identified synergism of these stressors and other measures of health, such as cardiovascular mortality (Anenberg et al. 2020).

Another important consideration is the heterogeneity of migraine disease causes and the spectrum of effects they can have on each individual (Marmura 2018). As such, the best interpretation of the given data may be that a certain subset of people with migraine is temperature sensitive, exhibiting a heterogeneity of trigger effects. Indeed, the two articles that specifically examined the effects of temperature at an individual level demonstrated significance in a subset of people with migraine; one of those articles lost that significance when studied in the whole population (Hoffmann et al. 2015).

The major limitations of this study are the low number of included studies and the inconsistency of measurements between them. More controlled studies are needed to investigate the sole effect of temperature on migraine disease, mitigating the presence of potential confounders or effect modifiers. Additionally, the authorial team included studies that investigate the relationship between temperatures among multiple headache disorders. The inclusion of these studies limits the specificity of conclusions reached in this investigation and affects the overall generalizability. Although focused explanations were conducted on the subset of people with migraine, further investigation, including only people with migraine, would provide additional strength.

The heterogeneity of the study designs introduced multiple confounders that could affect the interpretability and generalizability of results. Some of these range from differences within geographical areas, climate, barometric pressure, humidity, and air pollution. Further examples are differences in migraine diagnosis criteria across studies that may include people with migraine who might not fit the criteria utilized in other studies. These confounders were detailed in Table 2 for each included study.

**TABLE 2:** Description of confounding variables for all included articles.

Article	Air pollution	Humidity	Barometric pressure	Geography	Climate/weather change
Gomersall and Stuart 1973		+			+
Wilkinson and Woodrow (1979)		+	+		+
De Matteis et al. (1994)		+		+	+
Villeneuve et al. (2006)			+	+	+
Mukamal et al. (2009)	+		+		+
Zebenholzer et al. (2011)			+	+	+
Hoffmann et al. (2011)		+	+	+	+
Scheidt et al. (2013)				+	+
Hoffmann et al. (2015)		+	+	+	+
Yilmaz et al. (2015)		+	+	+	+
Yang et al. (2015)				+	+
Lee et al. (2018)	+			+	+

A limitation of all included studies is that outdoor air temperature cannot be controlled, as weather includes other elements such as pollution, humidity, and weather patterns. Thus, the association between temperature changes and migraine attack incidence does not mean that temperature changes cause more migraines. Similarly, the association between high temperatures and the increased association between pollution and migraine attack incidence does not demonstrate that heat causes pollution to cause more migraines.

Molecular mechanisms associated with temperature have also been associated with migraine attacks, such as butanoate and propionate, along with dysregulation of glycolysis/gluconeogenesis (Castor et al. 2021; Del Moro et al. 2022; Lanza et al. 2021, 2022; Martins‐Oliveira et al. 2017; Miao et al. 2022; Nassan et al. 2021; Okada et al. 1998; Peterlin et al. 2015; Ren et al. 2018; Shaw et al. 1977). There is no literature investigating the relationship between such molecular mechanisms in clinical settings. As such, further studies are needed to better characterize such relationships and could become potential therapeutic avenues for those patients living with migraine who are temperature sensitive.

Further overall research is needed to better characterize this relationship, especially comparing those who are temperature‐sensitive and those who may not be. Temperature and varying interpretations of the interplay of different ambient temperatures, such as indoor versus outdoor, create a very large space for potential observational and analytical studies. A potential experimental design could be to conduct a long‐term experiment for people with migraine in the same physical location to control for factors such as humidity. Such investigations could include the effect of indoor temperature or thermostat settings during both the day and night to further stratify effects of temperature based on environment. Trials could be conducted by separating people with migraine into low, moderate, and high indoor temperature groups and determining if there is any effect on migraine disease incidence.

## 5 Conclusion

The relationship between temperature and migraine incidence remains unclear. A slim majority of studies in this review demonstrated some type of relationship between temperature and migraine, but those relationships were varied and inconsistent. As such, further comparative studies looking at individual‐level effects while controlling for ambient temperature confounders are needed to further elucidate this relationship.

## Author Contributions

**James Kelbert**: conceptualization, writing – review and editing, writing – original draft, investigation, methodology, software, validation, visualization. **Joshua A. Tobin**: Conceptualization, supervision, writing – original draft, writing – review and editing, investigation.

## Conflicts of Interest

Joshua A. Tobin has no disclosures relevant to this submission but, in efforts to be fully transparent, has a significant other who currently works for Amgen, which owns Aimovig/erenumab. The other authors declare no conflicts of interest.

## Peer Review

The peer review history for this article is available at https://publons.com/publon/10.1002/brb3.70708

## Data Availability

Data will be made available upon request to the authors.
